# Isolation and characterization of phosphate-solubilizing bacteria from rhizosphere of poplar on road verge and their antagonistic potential against various phytopathogens

**DOI:** 10.1186/s12866-023-02953-3

**Published:** 2023-08-15

**Authors:** Zeng Qingwei, Tang Lushi, Zhang Yu, Shao Yu, Wu Wanting, Wang Jiangchuan, Ding Xiaolei, Han Xuejiao, Muhammad Bilal

**Affiliations:** 1 School of Life Science and Food Engineering, Huaiyin Institute of Technology, Huaian, 223003 China; 2 School of Accounting, Jiangsu Vocational College of Finance & Economics, Huaian, 223003 China; 3 Co-Innovation Center for Sustainable Forestry in Southern China, College of Forestry, Nanjing Forestry University, Nanjing, 210037 China

**Keywords:** PS activity, Antagonistic activity, Biofertilizer, Biocontrol, Poplar

## Abstract

**Background:**

Phosphate-solubilizing bacteria (PSB) can solubilize insoluble phosphate compounds and improve phosphate availability in soil. Road verges are important in urban landscaping, but the population structure of PSB and their ecological functions in the road verge soil is still unclear.

**Results:**

Twenty-one mineral PSB strains and 14 organic PSB strains were isolated from the rhizosphere of poplar on urban road verge. All the mineral PSB strains showed better solubilization to Ca_3_(PO_4_)_2_ than FePO_4_ or AlPO_4_. Among them, 7 strains showed high phosphate-solubilizing (PS) activities to Ca_3_(PO_4_)_2_ (150–453 mg/L). All the organic PSB strains displayed weak solubilization to lecithin. 16S rRNA gene-based phylogenetic analysis showed good species diversity of the PSB strains, which belongs to 12 genera: *Bacillus*, *Cedecea*, *Cellulosimicrobium*, *Delftia*, *Ensifer*, *Paenibacillus*, *Pantoea*, *Phyllobacterium*, *Pseudomonas*, *Rhizobium*, *Sinorhizobium* and *Staphylococcus*. Moreover, 8 PSB strains showed various degrees of growth inhibition against 4 plant pathogenic fungi, *Fusarium oxysporum* S1, *F. oxysporum* S2, *Pythium deliense* Meurs Z4, *Phomopsis* sp. AC1 and a plant pathogenic bacterium, *Pectobacterium carotovorum* TP1.

**Conclusions:**

The results indicated that these PSB strains could perform multiple ecological functions on road verge. The development and application of bio-agents based on the strains would provide a new strategy for maintaining and improving the ecosystem stability of road verges.

## Background

Phosphorus is one of the essential nutrients for the growth of plants. However, it’s also a growth-limiting nutrient in the soil due to its liability to fixation [1]. To overcome phosphate deficiency and improve available phosphate, a large scale of phosphate fertilizers have been applied in modern agriculture [2]. In a short time, enormous amounts of soluble phosphate (mainly phosphate ion) is brought into the soil by the application of phosphate fertilizers, but the utilization of the phosphate ion is very low because it’s easy to be fixed again by chelation with metal ions in soil [3]. Long periods and repeated applications of phosphate fertilizers not only raise environmental problems [4] but also compromise the soil micro-ecosystem balance, resulting in the loss of soil activities [5]. Fortunately, alternative and sustainable ways to solve soil phosphate deficiency are emerging with the discovery and research of phosphate-solubilizing microorganisms (PSM).

Phosphate-solubilizing microorganisms are regarded as typical plant growth-promoting rhizobacteria (PGPR) that can transform insoluble phosphate into soluble form and promote plant growth [5]. As an important component of PSM, PSB show good plant growth-promoting abilities and excellent application potential as biofertilizers [6]. Based on the types of phosphate substrates, they can be divided into mineral (inorganic) and organic PSB [7]. The solubilization of mineral phosphate source by mineral PSB mainly involves the secretion of organic acids, accompanied by a drop in pH value, and the solubilization (mineralization) of organic phosphate source relies on catalytic activities of phosphatase, phytase, or carbon-phosphorus lyase [8]. Isolation and screening of efficient PSB are the basis for biofertilizers, enhancing plant growth and crop yield [9]. The distribution of PSB in soil shows a significant rhizosphere preference in the population [10]. To date, amounts of PSB have been isolated from different rhizospheric soil, such as rice [11], maize [12], pea [13], papaya [14], et al.

Improving phosphate availability in soil is regarded as the primary feature of PSB for plant growth. Besides, the PSB also perform potential ecological functions in soil. The interaction of inoculated PSB and indigenous microorganisms would adjust the microbial diversity and composition in the rhizosphere in favour of plants [15, 16]. Antagonism against plant disease is one of the important ecological functions of PGPR [17]. More and more research indicates that some PSB possess potent antagonistic activities. For instance, tea rhizospheric PSB strain *Serratia marcescens* Pt-3 showed antagonism against seven different pathogenic fungi [18]. Inoculation with PSB strain *Burkholderia* sp. ‘N3’ could significantly reduce the incidence of disease caused by pathogenic bacteria [19]. Therefore, the antagonistic activity is likely to expand further the application potential of PSB in agriculture and forestry.

Road verges are a vital part of urban landscaping, which have ecological and aesthetic functions in the cities [20]. However, urban road verges’ ecosystem is always more fragile than natural forests because of its low biodiversity in plant species [21] and the special soil properties influenced by urban construction and traffic [22]. Moreover, the special conflicts of plants and their roots with city infrastructure elements also limit plant growth [23]. It is reported that the soil microorganisms play important roles in maintaining soil activity and plant adaptation to stresses [24]. Nevertheless, the contribution of the plant growth-promoting rhizobacteria (PGPR), especially PSB, to the ecological stability of the urban road verge is still unclear.

Poplar is the most common plant on road verges in China. In order to figure out the phosphate-solubilizing bacteria community and their functions in urban road verge soil, we isolated and screened mineral and organic PSB strains from the poplar rhizosphere on the road verge. The isolated PSB strains were identified, and their PS activities were evaluated. The antagonistic activities of the PSB strains against five phytopathogens were assessed. This study would facilitate understanding the population structure and functions of PSB strains in rhizospheric soil of the urban road verge.

## Methods

### Sample collection and isolation of PSB

The soil samples were collected from the rhizosphere of 15 years old poplar trees from a road verge in the city of Huaian, China (119.05’N, 33.57’E) in June, 2018. Three soil samples were collected from three different poplar trees. For obtaining the rhizospheric soils, surface soils were removed at a distance of 2–3 m to the trunk of the poplar trees. The soil samples were collected from a 6–15 cm layer close to the poplar roots. The sample shovel was sterilized with ethanol solution (75%) and sealed in a sterile self-sealing bag. The physical and chemical characteristics of the soil samples were analyzed by Anhui Pince Testing Technology Service Co. LTD. The contents of the total nitrogen, total phosphorus, total potassium, available phosphate, calcium, ferrum, magnesium, organic matter, and soil pH value were determined following the soil testing methods of the Agricultural Industry Standard of the People’s Republic of China. The National Botanical Research Institute’s Phosphate (NBRIP) growth agar medium and Pikovskaya’s agar media were used to isolate the mineral and organic PSB strains, respectively. Ten grams (fresh weight) of the soils were suspended in 100 mL of sterile saline solution and shaken at 150 rpm, 28 °C for 30 min in a rotary shaker. The mixed liquid was serially diluted into 10^− 6^-fold, and 100 µL of the diluent of 10^− 2^-10^− 6^ fold was plated on NBRIP and Pikovskaya’s agar media, respectively. After 5 days of stationary incubation at 28 °C, the colonies with a clear halo around were selected and streaked onto new LB agar media. After two days of incubation, the single colony on LB agar media was picked up with an inoculating loop and cultured in LB broth at 180 rpm, 28 °C for 16–24 h. The phosphate solubilization of the purified colonies was reconfirmed by inoculating 10 µL of the cultured media (10^8^ CFU/mL) into NBRIP or Pikovskaya’s agar media with three replicates respectively. The preliminary assessment of PS activities was evaluated by the ratio (R) (R = clear halo diameter/colony diameter). The isolated PSB strains were stored at -80 °C for further research.

### Assessment of the solubilizing activities of PSB strains

The NBRIP and Pikovskaya’s broth media were used to quantitatively evaluate the PS activities of the selected mineral and organic PSB strains, respectively. For mineral PSB, individually, Ca_3_(PO_4_)_2_, FePO_4_ and AlPO_4_ were used as insoluble phosphate sources in NBRIP broth media. After being cultured at 180 rpm, 28 °C for 16–24 h, the PSB strains were washed twice, diluted into 10^8^ CFU/mL with distilled water, and acted as inocula. Five hundred µL of the inoculum was inoculated into 50 mL NBRIP or Pikovskaya’s broth medium in a 100-mL Erlenmeyer flask, and the flasks were cultured at 180 rpm, 28 °C for 72 h. Each PSB strain was inoculated in triplicate, and the medium without inoculation served as a control. After incubation, the supernatant was separated by centrifugation at 10,000 g for 10 min and then filtrated with 0.22-mm-pore-size of medical millex-GP filters (Millipore, Bedford, Mass.). The concentration of solubilized phosphate in the supernatant was detected by using the ascorbate method [25]. The pH value was measured by a basic pH meter.

### 16 S rRNA gene and phylogenetic analysis

The PSB strains were incubated in LB nutrient broth overnight at 180 rpm, 28 °C and 1 mL of the culture medium of each PSB strain was centrifuged at 5000 rpm for 2 min, washed three times with sterile saline solution for bacterial cell collection. The collected bacterial cell was used for total genomic DNA isolation by the CTAB method [26]. The 16S rRNA genes were amplified by PCR using the total genomic DNA as template and universal bacterial primers (24 F: AGAGTTTGATCCTGGCTCAG and 1492R: TACGGYTACCTTGTTACGACTT) as primers. The PCR products were purified by using DNA purification kit (Axygen, USA) and sequenced by General Biol (Anhui) Co. LTD. The similarity of the 16S rRNA gene sequences was compared with the genetic database available using both the NCBI Blastn program (http://www.ncbi.nlm.nih.gov) and Eztaxon 16S-based Identify System (http://147.47.212.35:8080/). The identified sequences were uploaded to the GenBank nucleotide sequence database. The phylogenetic dendrogram was constructed by the neighbor-joining method and tree topology was evaluated by performing bootstrap analysis with 1,000 replicates using Molecular Evolutionary Genetics Analysis (MEGA 7.0) software.

### Antagonism against pathogenic microorganisms

The antagonistic activities of the PSB strains were tested by using confrontation or dual culture tests [27]. Five phytopathogens including four fungi: *F. oxysporum* S1, *F. oxysporum* S2, *P. deliense* Meurs Z4, *Phomopsis* sp. AC1 and one bacterium, *P. carotovorum* TP1, were selected in this study. The 4 pathogenic fungi were cultured on PDA agar plates under 28 °C. After one week of incubation, the PDA agar covered with the fungus hypha was cut into 6 mm diameter discs with a cork borer, and the discs were inoculated onto the center of a new PDA plate (Diameter 90 mm). The PSB strain inocula (100 µL) were inoculated to both sides of the pathogen discs at an equal distance. For the pathogenic bacterium *P. carotovorum* TP1, after incubation in LB nutrient broth overnight at 180 rpm, 28 °C, 150 µL of the culture medium with a concentration of 10^8^ CFU/mL was inoculated onto the center of a new LB plate, and the PSB strain inocula (100 µL) were inoculated to both sides of the pathogenic bacterium. The inoculated agar plates were incubated at 28 °C for 5 days. Each PSB strain vs. each pathogen strain was carried out in triplicate, and the agar plate inoculated with the pathogen strain acted as a control. The shortest diameter (d) of the pathogen colony that grew towards the bacterial colony on each confrontation plate and the maximum diameter (D) of the colony that grew away from the bacterial colony were measured after incubation. The antagonistic activity was quantified by visible zones of fungal growth inhibition (FGI) using the Eq. 1:

Antagonistic activity (%) = (D - d) / D × 100% (Eq. 1).

After the direct confrontation, the PSB strain with good performance in inhibiting pathogen growth was cultured in LB broth medium at 180 rpm, 28 °C for 24 h. The medium was centrifuged at 10,000 rpm for 3 min, and then the supernatant was filtrated with 0.22-mm-pore-size of medical millex-GP filters (Millipore, Bedford, Mass). The antagonistic activity of the supernatant was detected using the same direct confrontation method. One hundred µL of the supernatant was injected onto the both sides of the pathogen. The other operations were the same as above.

### Data Analysis and Processing

Microsoft Excel 2016 (Microsoft Corporation, Redmond, USA) was used to collate and analyze the data of PS activities. Multiple comparisons and correlation analyses of the data in the present study were carried out using SPSS (version 16.0) (IBM Inc., New York, USA) with the LSD method and Pearson method, respectively.

## Results

### Soil sample and PSB isolates

**Fig. 1:**
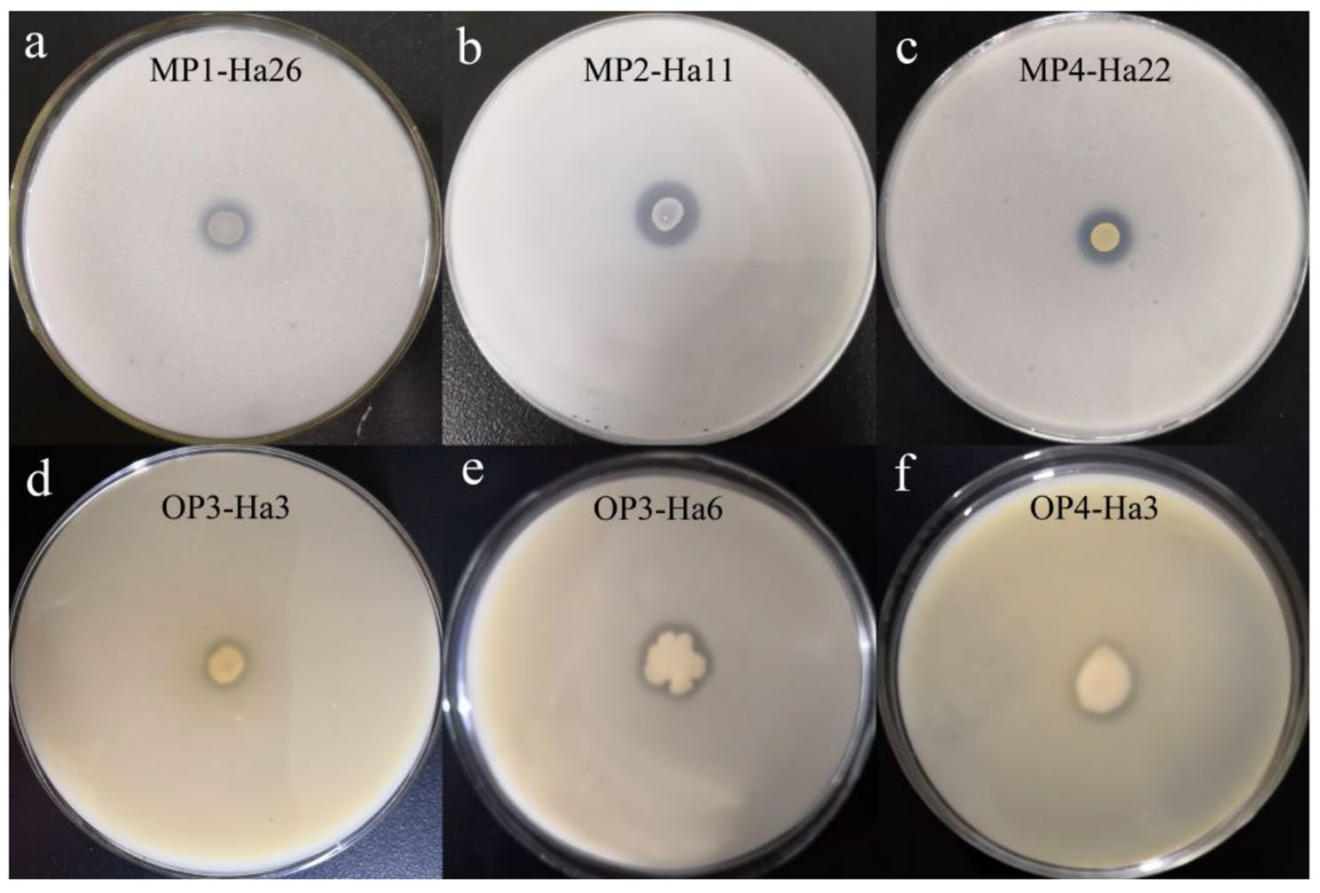
Preliminary assessments of PS activities with clear zone formation by the mineral PSB strains (a-c) and the organic PSB strains (d-f)

The chemical characteristics of the soil samples are displayed in Table 1. The soil is weakly alkaline. The total nitrogen and phosphorus amounts in soil are much lower than that of total potassium. The content of available phosphate was just 0.93% of the total phosphorus. For metallic elements, the amounts of Ca is higher than that of Fe and Mg. Besides, the organic matter is only 13.9 g/Kg soil. In the preliminary screening, 21 mineral PSB strains and 14 organic PSB strains showed obvious clear halo around their colonies on agar media, respectively. The ratio R of nine mineral PSB strains was beyond 1.5, and the ratio R of none of the organic PSB was beyond 1.5 (Table 2; Fig. 1).

**Table 1:** Physicochemical properties of the soil samples

Soil type	pH value	Total N (g/kg)	Total P (g/kg)	Total K (g/kg)	Available P (mg/kg)	Ca (g/kg)	Fe (g/kg)	Mg (g/kg)	Organic matter (g/kg)
Yellow	7.71 ± 0.10	0.516 ± 0.004	0.34 ± 0.06	15.8 ± 1.1	3.15 ± 0.27	3.53 ± 1.01	0.124 ± 0.009	0.73 ± 0.65	13.9 ± 1.2

Data are means of three replicates ± S. D

**Table 2:** Preliminary screening results of the PSB isolates

Mineral PSB isolates	Clear halo diameter / mm	Colony diameter / mm	Ratio R	Organic PSB isolates	Clear halo diameter / mm	Colony diameter / mm	Ratio R
Mp1-Ha1	13.9 ± 0.30	8.3 ± 0.42	1.67 ± 0.04^d^	Op1-Ha1	13.2 ± 1.45	12.1 ± 0.39	1.09 ± 0.03^cde^
Mp1-Ha3	14.9 ± 0.12	7.1 ± 0.53	2.11 ± 0.14^a^	Op1-Ha2	9.8 ± 0.74	8.9 ± 0.41	1.10 ± 0.04^cde^
Mp1-Ha4	10.1 ± 0.17	5.5 ± 0.26	1.84 ± 0.06^c^	Op1-Ha3	23.8 ± 1.13	21.1 ± 0.95	1.13 ± 0.02^bcd^
Mp1-Ha7	10.0 ± 0.17	4.9 ± 0.12	2.04 ± 0.02^ab^	Op1-Ha4	13.3 ± 0.64	12.3 ± 0.63	1.08 ± 0.02^de^
Mp1-Ha8	7.1 ± 0.51	4.6 ± 0.35	1.54 ± 0.01^e^	Op2-Ha3	11.1 ± 0.63	10.5 ± 0.76	1.06 ± 0.05^e^
Mp1-Ha10	10.0 ± 0.29	5.8 ± 0.37	1.72 ± 0.06^d^	Op3-Ha3	9.4 ± 0.73	8.2 ± 0.95	1.15 ± 0.09^bc^
Mp1-Ha11	10.5 ± 0.38	7.2 ± 0.15	1.46 ± 0.02^ fg^	Op3-Ha4	17.3 ± 0.98	15.3 ± 0.33	1.13 ± 0.07^ cd^
Mp1-Ha26	10.0 ± 0.40	8.0 ± 0.56	1.25 ± 0.04^j^	Op3-Ha5	13.4 ± 1.52	11.2 ± 0.45	1.20 ± 0.03^ab^
Mp1-Ha27	5.7 ± 0.37	5.7 ± 0.40	1.01 ± 0.01^ L^	Op3-Ha6	13.4 ± 0.63	12.5 ± 0.72	1.07 ± 0.01^de^
Mp1-Ha32	9.8 ± 0.23	5.0 ± 0.17	1.96 ± 0.02^b^	Op4-Ha2	10.6 ± 0.97	9.5 ± 0.56	1.12 ± 0.02^ cd^
Mp2-Ha4	6.3 ± 0.29	6.2 ± 0.31	1.01 ± 0.00^ L^	Op4-Ha3	15.7 ± 1.02	13.9 ± 0.64	1.13 ± 0.03^bcd^
Mp2-Ha8	7.5 ± 0.13	5.8 ± 0.22	1.29 ± 0.02^ij^	Op4-Ha4	16.7 ± 0.1.88	15.6 ± 0.95	1.07 ± 0.05^de^
Mp2-Ha11	13.5 ± 0.32	8.1 ± 0.45	1.67 ± 0.05^d^	Op4-Ha6	12.9 ± 1.68	10.3 ± 0.67	1.25 ± 0.03^a^
Mp2-Ha20	12.3 ± 0.10	8.4 ± 0.14	1.46 ± 0.02^ fg^	Op4-Ha7	15.9 ± 0.98	14.3 ± 0.85	1.11 ± 0.03^ cd^
Mp2-Ha21	7.7 ± 0.28	5.7 ± 0.36	1.35 ± 0.04^hi^				
Mp3-Ha1	9.6 ± 0.40	6.6 ± 0.22	1.45 ± 0.01^ fg^				
Mp4-Ha1	11.9 ± 0.38	8.4 ± 0.44	1.41 ± 0.03^gh^				
Mp4-Ha6	11.5 ± 0.17	7.6 ± 0.25	1.51 ± 0.03^ef^				
Mp4-Ha22	9.9 ± 0.33	8.0 ± 0.47	1.23 ± 0.03^jk^				
Mp4-Ha28	8.3 ± 0.19	6.6 ± 0.32	1.25 ± 0.03^j^				
Mp4-Ha30	7.4 ± 0.35	6.4 ± 0.21	1.16 ± 0.02^k^				

Data are means of three replicates ± S. D

Lowercase letters in the data of ratio R indicate significant differences at *P < 0.05* level.

### Phosphate solubilizing activities of the PSB isolates

**Fig. 2:**
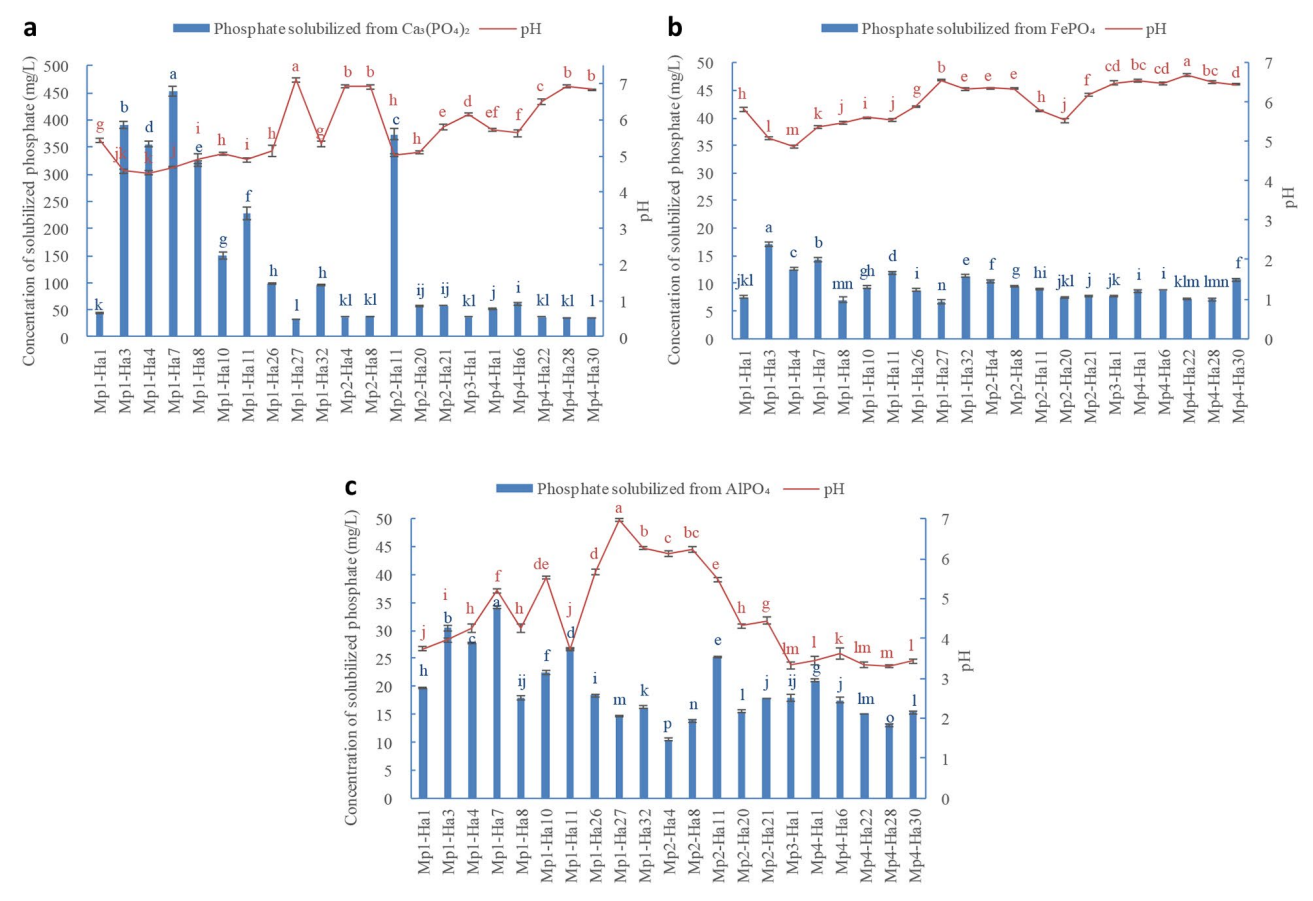
Phosphate-solubilizing activities of the mineral PSB strains and pH in the media. (**a**) Ca_3_(PO_4_)_2_ as mineral phosphate source, (**b**) FePO_4_ as mineral phosphate source, and (**c**) AlPO_4_ as mineral phosphate source. Error bars = S. (**D**). Lowercase letters above the bars indicate significant differences of the PS activities of different isolates and the media pH values at *P < 0.05* level, respectively

The PS activities of the 21 mineral PSB strains and 14 organic PSB strains were quantitatively assessed (Table 3). All the 21 mineral PSB strains showed much better solubilization to Ca_3_(PO_4_)_2_ than FePO_4_ or AlPO_4_. The Ca_3_(PO_4_)_2_ solubilizing activities of the strains presented positive correlation with their FePO_4_ (R = 0.649, *P < 0.01*) and AlPO_4_ (R = 0.857, *P < 0.01*) solubilizing activities. The concentrations of solubilized phosphate from Ca_3_(PO_4_)_2_ ranged from 33.14 to 453.33 mg/L, which were 6.69 to 17.08 mg/L for FePO_4_ and 13.02 to 34.16 mg/L for AlPO_4_. The concentrations of solubilized phosphate were the highest for Mp1-Ha7 (453.33 mg/L), Mp1-Ha3 (391.50 mg/L), Mp2-Ha11 (373.79 mg/L), Mp1-Ha4 (355.78 mg/L), Mp1-Ha8 (322.40 mg/L), Mp1-Ha11 (227.98 mg/L) and Mp1-Ha10 (150.36 mg/L) with Ca_3_(PO_4_)_2_ as the insoluble phosphate source. These results were consistent with the clear halo performances on NBRIP agar. The medium pH values decreased in varying degrees whiling the PSB strains solubilized the three insoluble phosphate sources (Fig. 2). The medium pH value was significantly negatively correlated with the concentration of solubilized phosphate when using Ca_3_(PO_4_)_2_ as the insoluble phosphate source (R=-0.904, *P < 0.05*), whiling it did not show any obvious relationship when using FePO_4_ or AlPO_4_ as the insoluble phosphate source. For organic PSB strains, the highest concentrations of solubilized phosphate were 9.07 mg/L for Op4-Ha3 and 2.237 mg/L for Op1-Ha2.

**Table 3:** Phosphate-solubilizing activities of the PSB strains

Mineral PSB isolates	Solubilized phosphate concentration (mg/L)			Organic PSB isolates	Solubilized phosphate concentration (mg/L)
	Ca_3_(PO_4_)_2_	FePO_4_	AlPO_4_		Lecithin
Mp1-Ha1	43.40 ± 0.98^k^	7.46 ± 0.29^jkl^	19.77 ± 0.11^ h^	Op1-Ha1	1.52 ± 0.04^c^
Mp1-Ha3	391.50 ± 0.26^b^	17.08 ± 0.34^a^	30.46 ± 0.48^b^	Op1-Ha2	2.23 ± 0.07^b^
Mp1-Ha4	355.78 ± 6.26^d^	12.61 ± 0.15^c^	27.82 ± 0.11^c^	Op1-Ha3	0.27 ± 0.03^j^
Mp1-Ha7	453.33 ± 9.63^a^	14.36 ± 0.36^b^	34.16 ± 0.24^a^	Op1-Ha4	0.51 ± 0.04^ h^
Mp1-Ha8	322.40 ± 5.03^e^	6.96 ± 0.52^mn^	17.96 ± 0.39^ij^	Op2-Ha3	1.32 ± 0.04^d^
Mp1-Ha10	150.36 ± 5.64^ g^	9.25 ± 0.28^gh^	22.45 ± 0.28^f^	Op3-Ha3	0.95 ± 0.08^e^
Mp1-Ha11	227.98 ± 11.05^f^	11.92 ± 0.29^d^	26.57 ± 0.23^d^	Op3-Ha4	0.76 ± 0.04^f^
Mp1-Ha26	98.54 ± 1.35^ h^	8.78 ± 0.17^i^	18.39 ± 0.20^i^	Op3-Ha5	1.00 ± 0.03^e^
Mp1-Ha27	33.14 ± 0.04^ L^	6.69 ± 0.42^n^	14.64 ± 0.20^ m^	Op3-Ha6	0.37 ± 0.04^i^
Mp1-Ha32	95.21 ± 1.12^ h^	11.25 ± 0.26^e^	16.22 ± 0.30^k^	Op4-Ha2	0.67 ± 0.02^ fg^
Mp2-Ha4	37.02 ± 0.08^kl^	10.35 ± 0.26^f^	10.45 ± 0.23^p^	Op4-Ha3	9.07 ± 0.12^a^
Mp2-Ha8	37.71 ± 0.12^kl^	9.42 ± 0.24^ g^	13.82 ± 0.19^n^	Op4-Ha4	0.93 ± 0.06^e^
Mp2-Ha11	373.79 ± 10.24^c^	8.85 ± 0.09^hi^	25.28 ± 0.14^e^	Op4-Ha6	0.63 ± 0.07^ g^
Mp2-Ha20	56.08 ± 2.38^ij^	7.41 ± 0.21^jkl^	15.46 ± 0.24^ L^	Op4-Ha7	1.28 ± 0.06^d^
Mp2-Ha21	57.98 ± 0.74^ij^	7.72 ± 0.07^j^	17.86 ± 0.05^j^		
Mp3-Ha1	36.38 ± 0.006^kl^	7.65 ± 0.12^jk^	17.94 ± 0.51^ij^		
Mp4-Ha1	51.82 ± 0.35^j^	8.57 ± 0.27^i^	21.04 ± 0.28^ g^		
Mp4-Ha6	61.11 ± 1.66^i^	8.79 ± 0.08^i^	17.55 ± 0.55^j^		
Mp4-Ha22	37.35 ± 0.15^kl^	7.25 ± 0.12^klm^	15.08 ± 0.06^ lm^		
Mp4-Ha28	36.00 ± 0.08^kl^	7.05 ± 0.27^lmn^	13.02 ± 0.22^o^		
Mp4-Ha30	35.83 ± 0.07^ L^	10.67 ± 0.26^f^	15.35 ± 0.25^ L^		

Data are means of three replicates ± S. D

Lowercase letters indicate significant differences in each column at *P < 0.05* level

### Phylogenetic identification of the PSB strains

**Fig. 3:**
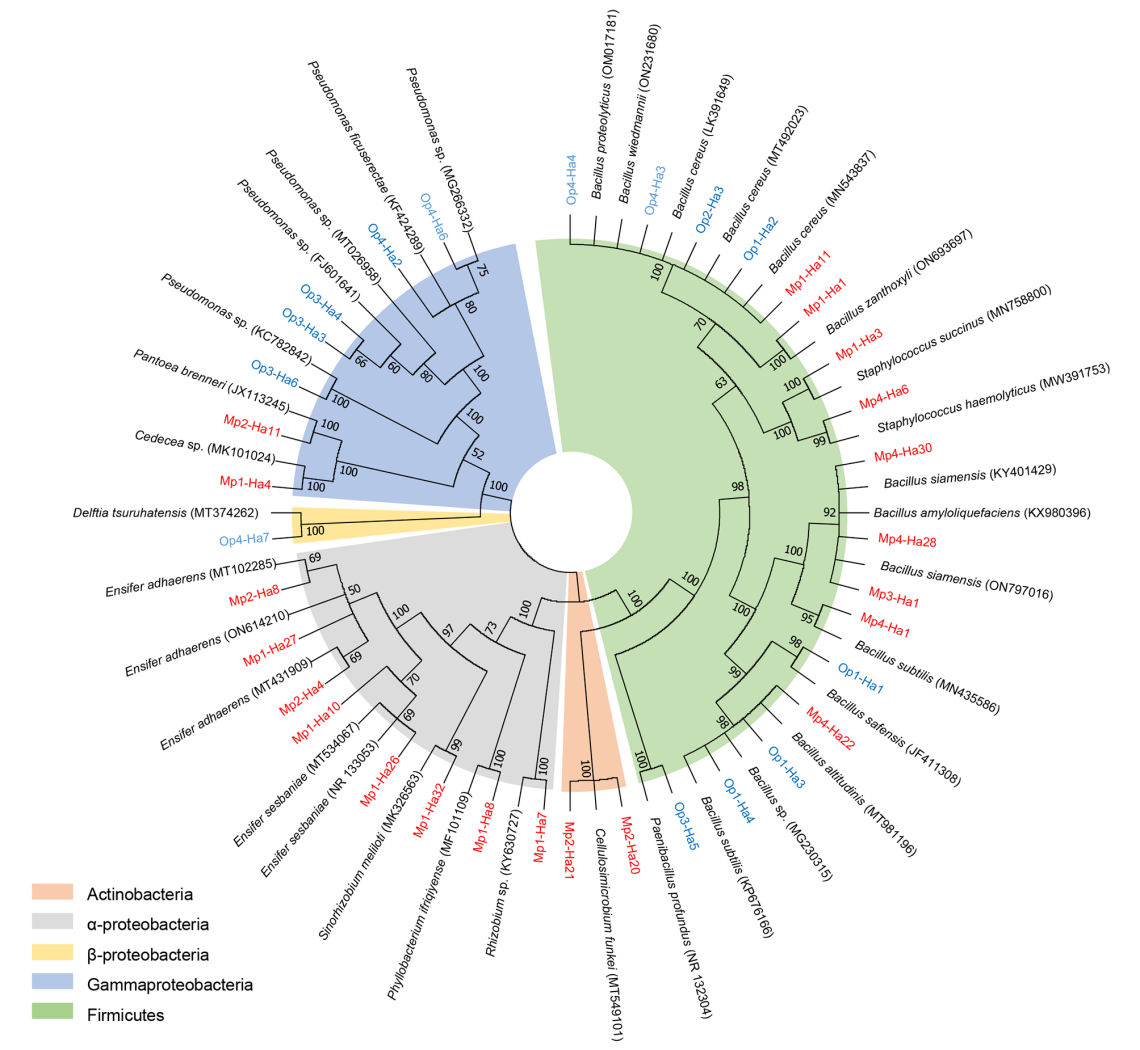
Phylogenic trees of the PSB strains based on the 16 S rRNA sequences. Maximum likelihood was used to construct the trees with bootstrapping (1000 replicates). The name of mineral PSB strains were marked in red, and the organic PSB strains were marked in blue

The maximum-likelihood phylogenetic trees based on the 16S rDNA sequences of the PSB strains and closest phylogenic reference strains were presented in Fig. 3. The 21 mineral PSB strains could be categorized into four groups: Firmicutes (42.9%), α-proteobacteria (38.1%), Gammaproteobacteria (9.5%), and Actinobacteria (9.5%). The 14 organic PSB strains could be categorized into three groups: Firmicutes (57.1%), Gammaproteobacteria (35.7%), and β-proteobacteria (7.1%). The α-proteobacteria and Actinobacteria on the tree were all mineral PSB strains and organic PSB strains were predominant in Gammaproteobacteria and β-proteobacteria. The Firmicutes distributed into mineral and organic PSB strains were around half to half (9/8). The identification of the PSB strains was combined with the alignments of the 16S rDNA sequences of the PSB strains and the closest phylogenic reference strains from the two databases. The accession numbers and the similarities results are listed in Table 4. The PSB strains belonged to 12 genera and 25 species. *Bacillus* (7 isolates) and *Ensifer* (5 isolates) were the most dominant genera for mineral PSB strains. *E. adhaerens* was the most dominant species (3 isolates). *Bacillus* (7 isolates) and *Pseudomonas* (5 isolates) were the most dominant genera for organic PSB strains. *Pseudomonas* sp. was the most dominant species (4 isolates).

**Table 4:** Alignment results of 16 S rDNA sequences of the PSB strains with genetic databases

Mineral PSB strains	Accession	GenBank database			EzBioCloud database		
		Top-hit strain	Identity	Accession	Top-hit taxon	Identity	Accession
			(%)			(%)	
Mp1-Ha1	OP003529	*Bacillus zanthoxyli*	99.86	ON693697	*Bacillus zanthoxyli*	99.58	KX865140
Mp1-Ha3	MN461567	*Staphylococcus succinus*	99.86	MN758800	*Staphylococcus succinus*	99.31	AF004220
Mp1-Ha4	MN461566	*Cedecea* sp.	100	MK101024	*Cedecea* sp.	98.84	CP009451
Mp1-Ha7	OP003530	*Rhizobium* sp.	99.92	KY630727	*Neorhizobium* sp.	98.71	SLYW01000027
Mp1-Ha8	OP003531	*Phyllobacterium ifriqiyense*	100	MF101109	*Phyllobacterium ifriqiyense*	100	AY785325
Mp1-Ha10	OP003532	*Ensifer sesbaniae*	99.85	MT534067	*Ensifer sesbaniae*	99.92	JF834143
Mp1-Ha11	OP003533	*Bacillus cereus*	99.86	MN543837	*Bacillus cereus*	99.93	AE016877
Mp1-Ha26	OP003534	*Ensifer sesbaniae*	99.85	NR_133053	*Ensifer sesbaniae*	99.71	JF834143
Mp1-Ha27	OP003535	*Ensifer adhaerens*	100	ON614210	*Ensifer adhaerens*	99.71	JNAE01000171
Mp1-Ha32	OP003536	*Sinorhizobium meliloti*	99.70	MK326563	*Sinorhizobium meliloti*	99.78	X67222
Mp2-Ha4	OP003537	*Ensifer adhaerens*	99.93	MT431909	*Ensifer adhaerens*	100	JNAE01000171
Mp2-Ha8	OP003538	*Ensifer adhaerens*	100	MT102285	*Ensifer adhaerens*	99.85	JNAE01000171
Mp2-Ha11	OP003539	*Pantoea brenneri*	98.45	JX113245	*Pantoea brenneri*	97.83	MIEI01000169
Mp2-Ha20	OP003540	*Cellulosimicrobium funkei*	100	MT549101	*Cellulosimicrobium funkei*	99.43	AY501364
Mp2-Ha21	OP003541	*Cellulosimicrobium funkei*	99.43	MT549101	*Cellulosimicrobium funkei*	99.29	AY501364
Mp3-Ha1	OP003542	*Bacillus siamensis*	100	ON797016	*Bacillus siamensis*	99.93	AJVF01000043
Mp4-Ha1	OP003543	*Bacillus subtilis*	99.86	MN435586	*Bacillus subtilis*	99.71	ABQL01000001
Mp4-Ha6	OP003544	*Staphylococcus haemolyticus*	99.72	MW391753	*Staphylococcus haemolyticus*	99.17	LILF01000056
Mp4-Ha22	OP003545	*Bacillus altitudinis*	99.93	MT981196	*Bacillus altitudinis*	100	ASJC01000029
Mp4-Ha28	OP003546	*Bacillus amyloliquefaciens*	100	KX980396	*Bacillus amyloliquefaciens*	99.71	FN597644
Mp4-Ha30	OP003547	*Bacillus siamensis*	100	KY401429	*Bacillus siamensis*	99.16	AJVF01000043
**Organic PSB strains**	**Accession**	**GenBank database**			**EzBioCloud database**		
		**Top-hit strain**	**Identity**	**Accession**	**Top-hit taxon**	**Identity**	**Accession**
			**(%)**			**(%)**	
Op1-Ha1	OP006269	*Bacillus safensis*	99.51	JF411308	*Bacillus safensis*	99.17	KY990920
Op1-Ha2	OP006270	*Bacillus cereus*	99.79	MT492023	*Bacillus cereus*	99.44	AE016877
Op1-Ha3	OP006271	*Bacillus* sp.	100	MG230315	*Bacillus* sp.	99.51	LJIY01000004
Op1-Ha4	OP006272	*Bacillus subtilis*	100	KP676166	*Bacillus* sp.	99.31	LJIY01000004
Op2-Ha3	OP006273	*Bacillus cereus*	99.93	LK391649	*Bacillus albus* (*cereus*)	99.58	MAOE01000087
Op3-Ha3	OP006274	*Pseudomonas* sp.	99.79	FJ601641	*Pseudomonas* sp.	99.23	CP011567
Op3-Ha4	OP006275	*Pseudomonas* sp.	99.86	MT026958	*Pseudomonas* sp.	99.37	CP011567
Op3-Ha5	OP006276	*Paenibacillus profundus*	99.51	NR_132304	*Paenibacillus profundus*	99.23	AB712351
Op3-Ha6	OP006277	*Pseudomonas* sp.	99.86	KC782842	*Pseudomonas* sp.	98.53	AP013068
Op4-Ha2	OP006278	*Pseudomonas ficuserectae*	99.93	KF424289	*Pseudomonas ficuserectae*	99.01	AB021378
Op4-Ha3	OP006279	*Bacillus wiedmannii*	100	ON231680	*Bacillus wiedmannii*	99.72	LOBC01000053
Op4-Ha4	OP006280	*Bacillus proteolyticus*	99.37	OM017181	*Bacillus proteolyticus*	99.44	MACH01000033
Op4-Ha6	OP006281	*Pseudomonas* sp.	99.86	MG266332	*Pseudomonas* sp.	99.43	CP017886
Op4-Ha7	OP006282	*Delftia tsuruhatensis*	99.86	MT374262	*Delftia tsuruhatensis*	99.22	BCTO01000107

### Antagonistic effect of the PSB strains against pathogenic microorganisms

**Fig. 4:**
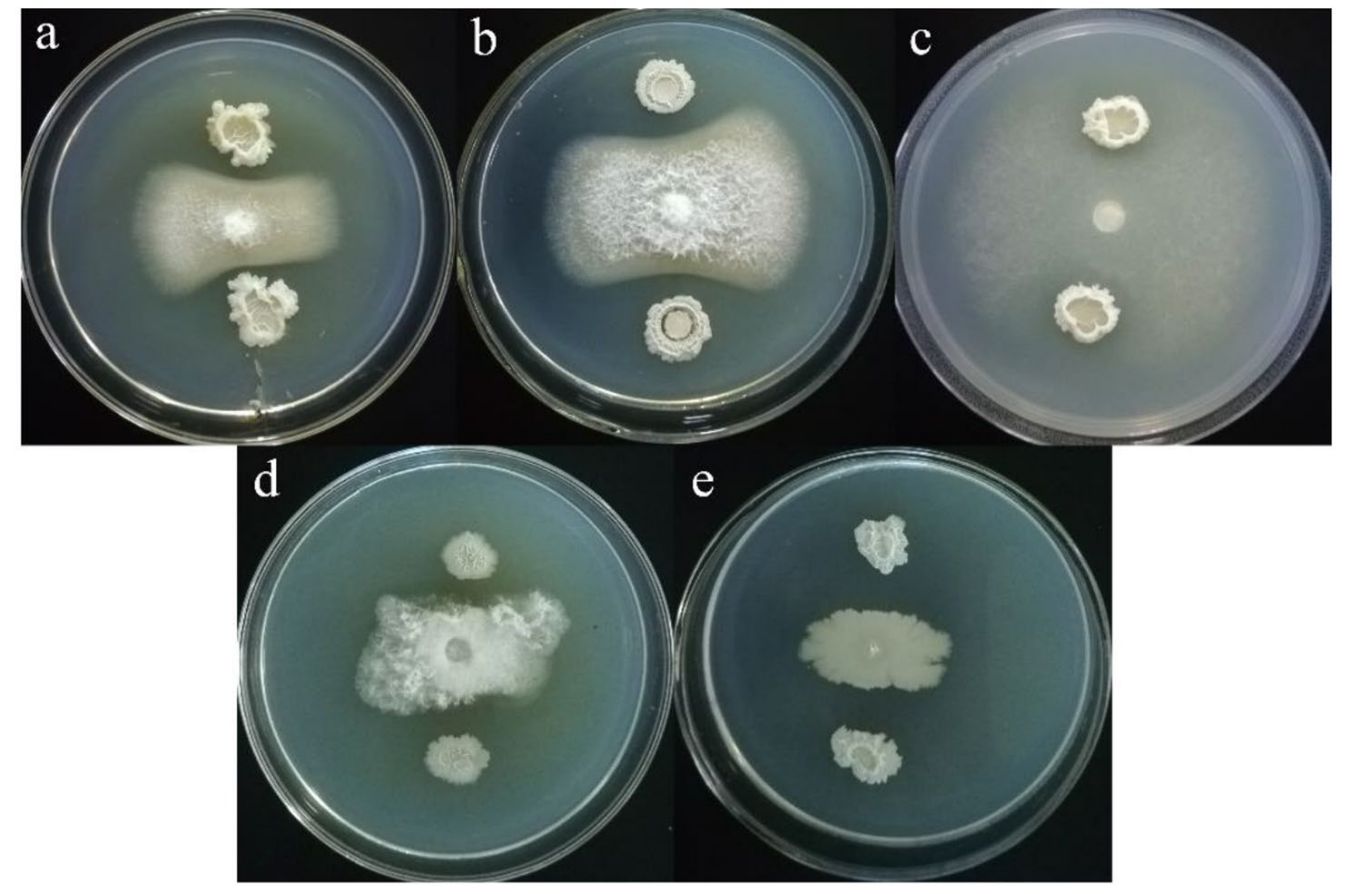
Antagonistic activities of the PSB strains against phytopathogen. (**a**) Growth of *F. oxysporum* S1 was inhibited by *B. amyloliquefaciens* Mp4-Ha28. (**b**) Growth of *F. oxysporum* S2 was suppressed by *B. siamensis* Mp4-Ha30. (**c**) Growth of *P. deliense* Meurs Z4 was prevented by *B. siamensis* Mp3-Ha1. (**d**) Growth of *Phomopsis* sp. A1 was repressed by *B. siamensis* Mp4-Ha30. (**e**) Growth of *P. carotovorum* TP1 was restrained by *B. amyloliquefaciens* Mp4-Ha28.

In the dual culture tests, 8 PSB strains showed obvious growth inhibition against the phytopathogens (Table 5; Fig. 4). The growth of phytopathogens *F. oxysporum* S1 and S2 were inhibited by *B. siamensis* Mp3-Ha1, *B. subtilis* Mp4-Ha1, *B. amyloliquefaciens* Mp4-Ha28, and *B. siamensis* Mp4-Ha30, among which, *B. amyloliquefaciens* Mp4-Ha28 and *B. siamensis* Mp4-Ha30 displayed the best antagonism to *F. oxysporum* S1 and S2 with FGI of 63.84 and 66.51%, respectively. Five PSB strains performed powerful antagonistic activities against *P. deliense* Meurs Z4, and *B. siamensis* Mp3-Ha1 showed the highest growth inhibition rate (FGI 78.21%). The growth of *Phomopsis* sp. A1 was only restrained by *B. siamensis* Mp4-Ha30 (FGI 65.32%). On the contrary, the growth of *P. carotovorum* TP1 could be inhibited by all 8 PSB strains, and the highest growth inhibition rate (FGI 55.86%) was carried out by *B. amyloliquefaciens* Mp4-Ha28. It is noticed that *B. siamensis* Mp4-Ha30 showed growth inhibition against all 5 phytopathogens (FGI > 40%). *B. zanthoxyli* Mp1-Ha1, *P. ifriqiyense* Mp1-Ha8, and *C. funkei* Mp2-Ha20 only performed growth inhibition against the *P. carotovorum* TP1.

Furthermore, the antagonistic activities of *B. siamensis* Mp3-Ha1, *B. subtilis* Mp4-Ha1, *B. amyloliquefaciens* Mp4-Ha28, and *B. siamensis* Mp4-Ha30 did not show any significant (*P < 0.05*) differences when used the supernatants as substitutes of the PSB strains in the dual culture tests. Nevertheless, *B. zanthoxyli* Mp1-Ha1, *P. ifriqiyense* Mp1-Ha8, *C. funkei* Mp2-Ha20 and *B. altitudinis* Mp4-Ha22 completely lost their growth inhibition activities to the phytopathogens when using the supernatants instead of the strain. Noticeably, no organic PSB strains presented antagonistic activities in the tests.

**Table 5:** Antagonistic activities of the PSB strains against phytopathogen

PSB Strains		FGI				
		*F. oxysporum* S1	*F. oxysporum* S2	*P. deliense* Meurs Z4	*Phomopsis* sp. A1	*P. carotovorum* TP1
*B. zanthoxyli* Mp1-Ha1	Strain	-	-	-	-	++
	Supernatant	-	-	-	-	-
*P. ifriqiyense* Mp1-Ha8	Strain	-	-	-	-	+
	Supernatant	-	-	-	-	-
*C. funkei* Mp2-Ha20	Strain	-	-	-	-	++
	Supernatant	-	-	-	-	-
*B. siamensis* Mp3-Ha1	Strain	++	+++	+++	-	++
	Supernatant	++	+++	+++	-	++
*B. subtilis* Mp4-Ha1	Strain	+++	++	+++	-	+
	Supernatant	+++	++	+++		++
*B. altitudinis* Mp4-Ha22	Strain	-	-	+++	-	+
	Supernatant	-	-	-	-	-
*B. amyloliquefaciens* Mp4-Ha28	Strain	+++	++	+++	-	++
	Supernatant	+++	++	+++	-	++
*B. siamensis* Mp4-Ha30	Strain	++	+++	+++	+++	++
	Supernatant	+++	+++	+++	+++	+++

FGI means fungal growth inhibition. “-” means no obvious inhibition of pathogenic microorganisms. “+” means FGI < 40%. “++” means 40% < FGI < 60%. “+++” means FGI > 60%

## Discussion

It is reported that the soil from road verges have lower levels of nutrients and humus compared to natural areas [22]. In this study, the contents of the nutrient elements nitrogen, phosphorus, potassium and organic matter in the road verge soil were below the average levels of natural soil in China (total nitrogen: 2.65–6.95 g/kg, total phosphorus: 0.44–0.85 g/kg, total potassium: 24.9–33.2 g/kg, organic matter: 40–50 g/kg) [28]. Less organic PSB strains were isolated than mineral PSB strains, which would be caused by the low concentration of humus in the soil [29]. Regular cleaning in the city removes most of the litter from road verges and blocks the decomposition of litter into organic phosphate and other organic matters in the soil [30]. The natural selection of a low level of organic phosphate would account for the low detection frequency of organic PSB and their low PS activities.

On the contrary, the mineral PSB strains in this study showed better performances both in isolated numbers and PS activities. The concentration of solubilized phosphate released from Ca_3_(PO_4_)_2_ by 7 strains was above 150 mg/L. The quantitative results of PS activities in broth media were consistent (78%) with the preliminary assessment on an agar plate, suggesting the reliability of the clear halo assessment of PS activity on NBRIP agar. It is demonstrated that the mineral PSB strains solubilize mineral phosphate by acidifying the environment with organic acids secretion [6, 31]. We also found a negative correlation between the PS activities and the media pH (R=-0.904, *P < 0.05*). Although the media pH also showed an obvious decrease when FePO_4_ or AlPO_4_ was use as a mineral phosphate source, the concentrations of solubilized phosphate released by the PSB strains from FePO_4_ or AlPO_4_ were much lower than that from Ca_3_(PO_4_)_2_. Similar results were also obtained by Illmer et al. [32, 33]. They thought the organic acids secretion and environment acidification were not the only mechanism for the PSB strain to solubilize mineral phosphate. Moreover, the nature of the organic acids significantly affects the mineral phosphate solubilization by PSB [34]. These would explain the variety of PS activities toward different mineral phosphate sources by the PSB strains.

Isolation and screening of PSB strains from different soil resources are the bases for further research and application of PSB as biofertilizers or biocontrol agents [5, 9]. Up to now, the PSB strains in road verges soils were rarely researched. In this study, the phylogenetic identification results showed that *Bacillus* (7 isolates), *Ensifer* (5 isolates) and, *Bacillus* (7 isolates), *Pseudomonas* (5 isolates) were the most dominant genera of mineral and organic PSB strains. The isolated strains belonged to the common categories of PGPR, like α-proteobacteria, β-proteobacteria, Actinobacteria, Firmicutes, and Gammaproteobacteria [35, 36]. Among them, *Bacillus* and *Pseudomonas* are two of the most abundant genera of PSB strains in diverse soil resources [11, 37, 38]. In the present study, the seven most effective PSB strains were *S. succinus* Mp1-Ha3, *Cedecea* sp. Mp1-Ha4, *Rhizobium* sp. Mp1-Ha7, *P. ifriqiyense* Mp1-Ha8, *E. sesbaniae* Mp1-Ha10, *B. cereus* Mp1-Ha11 and *P. brenneri* Mp2-Ha11. It displayed good microbial diversity of PSB strains in the road verge soil and provided good microbial resources for further PGPR research.

As a typical PGPR, PSB strains play important roles in adjusting the rhizospheric microbial community, providing a beneficial microecological environment for the plants [16]. Pathogenic resistance abilities of PSB strain, including bacteriostasis ability and antifungal activity, have been demonstrated to be one of the beneficial traits for the associated plant [18, 19]. This study assessed the antagonistic activities of PSB strains against different types of phytopathogens. *F. oxysporum* is a soil-colonized fungal phytopathogen with wide host range and strong pathogenicity [39]. In this study, 4 PSB strains showed growth inhibition performances against the soil fungal phytopathogens, *F. oxysporum* S1 and S2. *P. deliense* Meurs is regarded as a fungal phytopathogen from the rhizosphere, leading to root disease [40]. In this study, 5 PSB strains could inhibit the growth of *P.deliense* Meurs Z4. Unlike the soil fungal phytopathogen, the poplar canker disease pathogen *Phomopsis* sp. A1 can just be restained by *B. Siamensis* Mp4-Ha30. Previous study have shown that the effective biocontrol strain *B. pumilus* to poplar canker disease pathogen *P. macrospora* is isolated in the poplar stem, especially from the infected tissues [41]. Similarly, the biocontrol agent against the sheath blight pathogen of rice is also found in the rice tissues [42]. These results implied that the rhizosphere would not be the best place to isolate the biocontrol agents against the pathogen of aboveground diseases. As the only bacterial phytopathogen, *P. carotovorum* is also a soil phytopathogen that causes soft rot disease on many crops [43]. In the present study, the strain *P. carotovorum* TP1 was restrained by all 8 PSB strains. It is demonstrated that the growth of bacterial pathogens is easy to be suppressed by many secondary metabolites secreted by PGPR [44, 45]. Therefore, The secondary metabolites secreted by the PSB strains would be the main mechanism for the antagonistic activities against the strain *P. carotovorum* TP1.

Currently, the antifungal microorganisms seem to catch more attention, and the mechanisms of antagonism against fungal phytopathogen are more complicated [17, 27]. Overall, the mechanisms of biocontrol agents against the phytopathogens can be included as follows: (1) competing with pathogens; (2) production of extracellular antimicrobial metabolites, like antibiotics, lytic enzymes, hydrolytic enzymes, and bacteriocins. et al.; (3) priming plants for induced systemic resistance [45, 46]. In this study, the supernatants of some of the PSB strains also presented antagonistic activities with the strain tests. The extracellular metabolites would account for the antagonistic abilities of the PSB strains. The mechanisms of antagonism of these strains against the fungal phytopathogens still need further research in future.

## Conclusions

In this study, 21 mineral and 14 organic PSB strains belonged to 12 genera and 25 species were isolated from road verge soil. The 21 mineral PSB strains showed much better solubilization to Ca_3_(PO_4_)_2_ than FePO_4_ or AlPO_4_ and the pH values in the media decreased whiling the strains solubilized these insoluble mineral phosphate sources. The PS activities of 7 mineral PSB strains were above 150 mg/L in solubilizing Ca_3_(PO_4_)_2_. On the contrary, all the organic PSB strains presented weak solubilization to lecithin. In the dual culture tests, 8 strains exhibited antagonistic activities against 4 plant pathogenic fungi, *F. oxysporum* S1, *F. oxysporum* S2, *P. deliense* Meurs Z4, *Phomopsis* sp. AC1 and a plant pathogenic bacterium, *P. carotovorum* TP1. Among them, the strain *B. siamensis* Mp4-Ha30 presented growth inhibition to all the five phytopathogens (FGI > 40%) and all 8 PSB strains showed inhibition to the growth of the pathogenic bacterium, *P. carotovorum* TP1.

## Data Availability

The datasets used and/or analyzed during the current study are available from the corresponding author upon reasonable request.

## Abbreviations

PSM: Phosphate-solubilizing microorganism
PGPR: Plant growth-promoting rhizobacteria
PSB: Phosphate-solubilizing bacteria
PS activity: Phosphate-solubilizing activity
NBRIP: Botanical Research Institute’s Phosphate
FGI: Fungal growth inhibition
